# Strawberry leaf extract protects dopaminergic neurons by restoring redox and mitochondrial homeostasis

**DOI:** 10.3389/fnut.2026.1893670

**Published:** 2026-09-07

**Authors:** Chiharu Ueda, Tamaki Tokumatsu, Fuka Sogame, Nobuaki Chiba, Toshio Norikura, Yutaro Sasaki, Isao Matsui-Yuasa, Akiko Kojima-Yuasa

**Affiliations:** 1 Department of Nutrition, Graduate School of Human Life and Ecology, Osaka Metropolitan University, Osaka, Japan; 2 Department of Nutrition, Aomori University of Health and Welfare, Aomori, Japan

**Keywords:** AMPK, mitochondrial dysfunction, mitophagy, Nrf2, oxidative stress, Parkinson’s disease, strawberry leaf extract

## Abstract

**Introduction:**

Parkinson’s disease (PD) is a progressive neurodegenerative disorder characterized by the selective loss of dopaminergic neurons, for which effective disease-modifying strategies remain limited. Mitochondrial dysfunction and oxidative stress are central drivers of PD pathogenesis, highlighting the importance of cellular defense mechanisms targeting these processes.

**Methods:**

In the present study, we investigated the neuroprotective effects of strawberry leaf extract (SLE), an agricultural by-product rich in polyphenols, using both in vitro and in vivo models of PD.

**Results:**

In SH-SY5Y cells, SLE significantly attenuated rotenone-induced loss of cell viability and suppressed intracellular reactive oxygen species (ROS) production. Mechanistically, SLE promoted nuclear translocation of nuclear factor erythroid 2-related factor 2 (Nrf2) and increased the expression of antioxidant genes, including heme oxygenase-1 (HO-1) and p62. Pharmacological inhibition experiments further indicated that activation of AMP-activated protein kinase (AMPK) contributes to SLE-induced Nrf2 activation. In addition to redox regulation, SLE modulated mitochondrial quality control pathways. Time-dependent alterations in mitophagy-related markers, including PINK1, Parkin, LC3, and p62, were observed, accompanied by recovery of mitochondrial membrane potential. These findings suggest that SLE influences mitochondrial homeostasis under oxidative stress conditions. In a rotenone-induced PD mouse model, SLE administration ameliorated motor dysfunction and attenuated the loss of tyrosine hydroxylase-positive dopaminergic neurons in the substantia nigra.

**Discussion:**

Collectively, these results demonstrate that SLE exerts neuroprotective effects through coordinated regulation of the AMPK -Nrf2 signaling axis and mitochondrial quality control pathways. This study provides mechanistic insight into the potential of food-derived bioactive compounds as modulators of neurodegenerative processes and highlights SLE as a promising candidate for PD prevention or intervention.

## Graphical abstract

**Figure:**
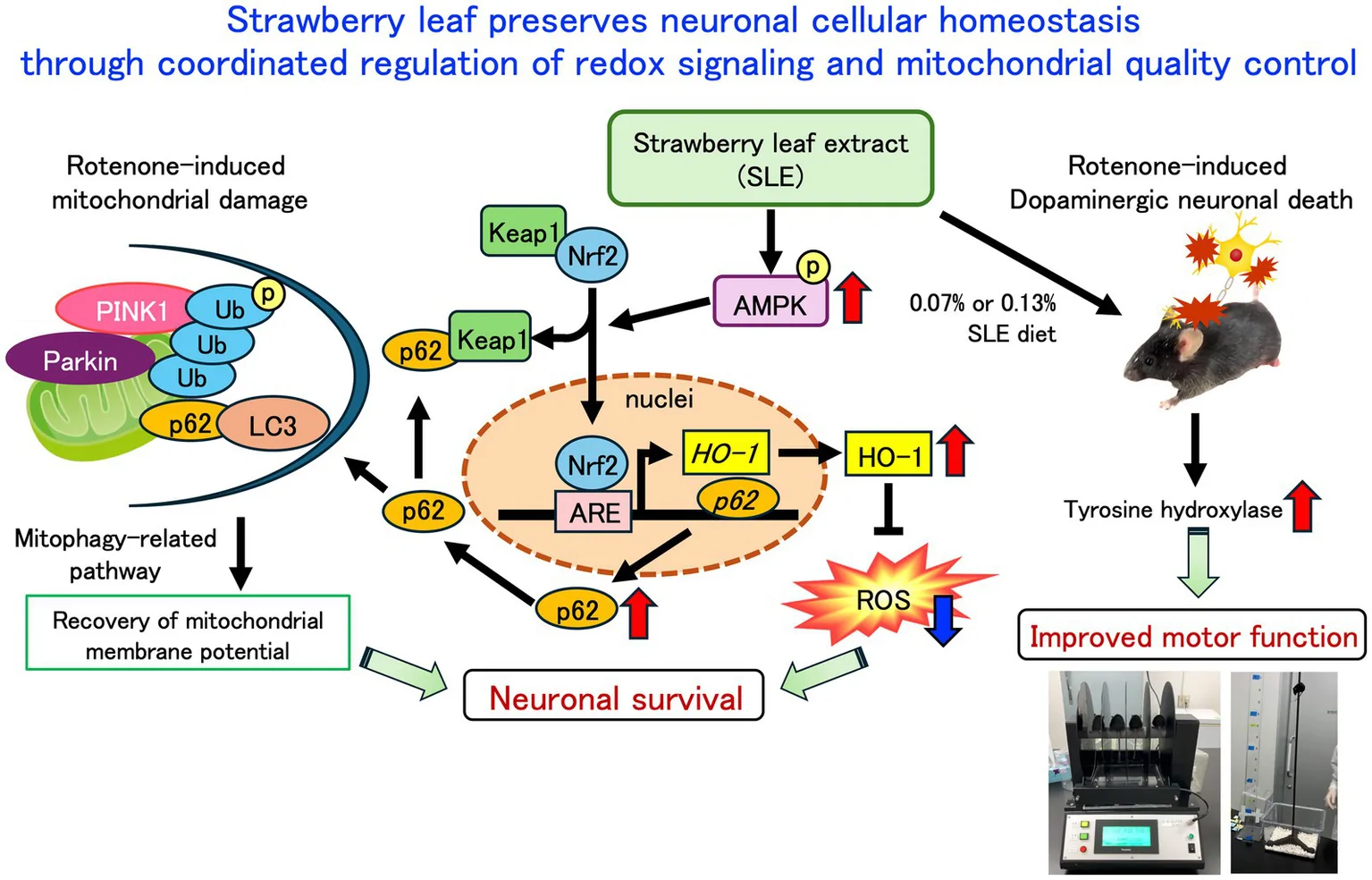
Proposal mechanism underlying the neuroprotective effect of Strawberry leaf extract.

## 1 Introduction

Parkinson’s disease (PD) is a progressive neurodegenerative disorder characterized by the selective loss of dopaminergic neurons in the substantia nigra, resulting in cardinal motor symptoms including resting tremor, rigidity, bradykinesia, and postural instability. In addition to motor dysfunction, PD is associated with diverse non-motor symptoms such as cognitive impairment, sleep disturbances, and psychiatric disorders, all of which substantially reduce quality of life (1). As the second most common neurodegenerative disorder after Alzheimer’s disease, the global prevalence of PD is rapidly increasing with population aging (2). The number of affected individuals is projected to exceed 14 million by 2040, raising major medical and socioeconomic concerns worldwide (3). Despite this growing burden, currently available therapies are largely symptomatic and do not effectively prevent or halt disease progression (4).

Accumulating evidence suggests that oxidative stress and mitochondrial dysfunction are central contributors to dopaminergic neurodegeneration (5, 6). Impairment of mitochondrial electron transport increases reactive oxygen species (ROS) production, leading to oxidative damage of cellular macromolecules and further deterioration of mitochondrial function (7). This vicious cycle ultimately disrupts neuronal homeostasis and promotes neuronal death. Accordingly, endogenous defense systems that coordinate redox regulation and mitochondrial maintenance have emerged as important targets for neuroprotective interventions.

The nuclear factor erythroid 2–related factor 2 (Nrf2) pathway plays a pivotal role in cellular adaptation to oxidative stress. Under basal conditions, Nrf2 is retained in the cytoplasm by Kelch-like ECH-associated protein 1 (Keap1) and undergoes proteasomal degradation (8). Upon oxidative or electrophilic stress, Nrf2 is stabilized and translocates into the nucleus, where it induces the expression of antioxidant and cytoprotective genes (9, 10). In addition to redox regulation, Nrf2 signaling has been linked to autophagy-related processes through regulation of p62, a multifunctional adaptor protein involved in the clearance of damaged cellular components, including mitochondria (11, 12). These findings suggest that coordinated regulation of redox balance and mitochondrial homeostasis may represent an effective strategy for protecting dopaminergic neurons.

Rotenone, a naturally occurring pesticide, is widely used as an experimental model of PD because it inhibits mitochondrial complex I, resulting in excessive ROS generation, mitochondrial dysfunction, and selective dopaminergic neuronal degeneration (13–15). Both *in vitro* and *in vivo* rotenone models reproduce key pathological features of PD, including loss of tyrosine hydroxylase-positive neurons and motor dysfunction (16, 17), and are therefore commonly employed to evaluate neuroprotective interventions.

Recently, increasing attention has been directed toward food-derived bioactive compounds as modulators of cellular stress responses. In particular, dietary polyphenols have been shown to exert neuroprotective effects not only through direct antioxidant activity but also through activation of endogenous defense pathways, including Nrf2 signaling (18). These observations have stimulated growing interest in nutritional approaches for the prevention of neurodegenerative disorders.

Strawberry (*Fragaria × ananassa* Duch.) leaves are an abundant agricultural by-product and are rich in polyphenols, including quercetin and kaempferol derivatives (19), often at levels higher than those found in the edible fruit (20). Recent studies have suggested antioxidant and neuroprotective-related properties of strawberry leaf extracts (21, 22). However, their potential protective effects against PD-related neuronal damage and the underlying mechanisms remain poorly understood. In particular, whether strawberry leaf extract (SLE) can modulate integrated cellular stress response pathways associated with redox regulation and mitochondrial homeostasis has not been clarified.

Therefore, the present study investigated the protective effects of SLE against rotenone-induced neuronal damage using both cellular and animal models of PD. We further examined the involvement of signaling pathways associated with redox regulation, energy sensing, and mitochondrial homeostasis in order to elucidate the mechanistic basis underlying the neuroprotective potential of SLE as a food-derived preventive strategy for PD.

## 2 Materials and methods

### 2.1 Preparation of strawberry leaf extract

Strawberry leaves (*Fragaria × ananassa* Duch.; cultivars Akihime, Benihoppe, Yotsuboshi, and a mixture of the three) were provided by Express Co., Ltd. (Japan). The leaves were freeze-dried, pulverized, and extracted with 50% (v/v) ethanol under continuous stirring at room temperature for 2 h. The extract was then stored at 4 °C overnight, filtered, concentrated under reduced pressure using a rotary evaporator, and lyophilized to obtain strawberry leaf extract (SLE). The total polyphenol contents of the 50% ethanol extracts from Akihime, Benihoppe, and Yotsuboshi, calculated as gallic acid equivalents, were 276.1 ± 8.9, 248.4 ± 16.8, and 218.4 ± 8.9 μg/mg extract, respectively. In the present study, SLE was characterized by its total polyphenol content, which was used as a practical index for extract standardization. Although strawberry leaves have been reported to contain diverse phenolic compounds, including ellagitannins, proanthocyanidins, flavonols, phenolic acids, and quercetin- and kaempferol-derived compounds (23), detailed qualitative and quantitative profiling of individual phenolics was not performed in the present study. Dried strawberry leaf powder was extracted using an in-house method developed in our laboratory. Briefly, the powder was mixed with 20 volumes of 50% ethanol and extracted by stirring at 100 rpm for 2 h. The mixture was then left overnight at 4 °C. On the following day, the extract was filtered through No. 3 filter paper, concentrated to dryness using a rotary evaporator, and subsequently lyophilized. The extraction yields of the “Akihime,” “Benihoppe,” and “Yotsuboshi” cultivars were 26.2, 29.5, and 27.4%, respectively.

The dried extract was stored at −80 °C and dissolved in dimethyl sulfoxide (DMSO; FUJIFILM Wako Pure Chemical Corporation, Osaka, Japan) at the time of use.

### 2.2 Cell culture

Human neuroblastoma SH-SY5Y cells (KAC Co., Ltd., Kyoto, Japan) were cultured in Dulbecco’s Modified Eagle’s Medium (DMEM; Shimadzu Diagnostics Corporation, Tokyo, Japan) supplemented with 10% fetal bovine serum (FBS) at 37 °C in a humidified atmosphere containing 5% CO₂. The medium was replaced every 2–3 days, and cells were subcultured at 70–80% confluence. The composition of DMEM is presented in Table 1. Cells between passage 21 and 30 were used in the experiments. The final DMSO concentration was adjusted to 0.1% (v/v) in all experimental and control groups.

**Table 1:** Composition of DMEM.

Components	(mg/L)
NaCl	6400.0
KCl	400.0
CaCl_2_	200.0
MgSO_4_	97.7
NaH_2_PO_4_	140.0
Fe(NO_3_)_2_·9H_2_O	0.1
d-Glucose	1000.0
Sodium pyruvate	110.0
Succinic acid	106.0
Disodium succinate (anhydrous)	16.2
l-Arginine·HCl	84.0
l-Cystein·HCl·H_2_O	70.3
Glycine	30.0
l-Histidine·HCl·H_2_O	42.0
l-Isoleucine	104.8
l-Leucine	104.8
l-Lysine·HCl	146.2
l-methionine	30.0
l-Phenylalanine	66.0
l-Serine	42.0
L-Threonine	95.2
l-Tryptophane	16.0
l-Tyrosine disodium	89.5
l-Valine	93.6
Choline bitartrate	7.2
Folic acid	4.0
Nicotinamide	4.0
Calcium pantothenate	4.0
Pyridoxal·HCl	4.0
Riboflavin	0.4
Thiamine·HCl	4.0
Myo-Inositol	7.2
Phenol red	5.0

DMEM was prepared by adding 0.1% penicillin, 0.1% streptomycin, and filter-sterilized 2% L-glutamine (584 mg/L) to autoclaved DMEM, followed by pH adjustment to 7.5 using sterile 8% NaHCO₃.

### 2.3 Cell viability assay

Cell viability was assessed using the MTT assay. SH-SY5Y cells were seeded in 96-well plates (2.0 × 10^4^ cells/well) and cultured overnight. Cells were treated with SLE (0–200 μg/mL) and/or rotenone (0–400 nM; Sigma-Aldrich Japan K. K., Tokyo, Japan) for 24 h.

After treatment, the medium was replaced with 100 μL of fresh medium containing 0.5 mg/mL MTT and incubated for 2 h at 37 °C. The medium was then removed and 200 μL of DMSO was added to each well to dissolve the formazan crystals. Absorbance was measured at 550 nm using a microplate reader (Wallac 1,420 ARVOsx, PerkinElmer, United States).

### 2.4 Measurement of intracellular ROS

Intracellular reactive oxygen species (ROS) levels were measured using 2′,7′-dichlorofluorescin diacetate (DCFH-DA; FUJIFILM Wako). Cells were incubated in serum-free medium with DCFH-DA (6 μM) during the final 30 min of a 6 h treatment period. After washing with PBS, fluorescence images were captured using an FSX100 fluorescence microscope (Olympus, Tokyo, Japan). Fluorescence was detected using the B-excitation filter set, consisting of a 460-495-nm band- pass excitation filter, BA a 510-550-nm barrier filter and a 505-nm dichroic mirror.

Fluorescence intensity was quantified using ImageJ software (version 1.54). For each image, the mean background fluorescence intensity measured at 10 cell-free regions was subtracted from the fluorescence intensity of each cell. Forty cells per group from 4 independent biological experiments were analyzed, and the mean background-corrected fluorescence intensity per cell was calculated.

### 2.5 Quantitative real-time PCR

Total RNA was extracted using the High Pure RNA Isolation Kit (Roche, Switzerland). RNA quality was assessed using an Agilent 2,100 Bioanalyzer. Total RNA was reverse-transcribed using the ReverTra Ace^®^ qPCR RT Master Mix with gDNA Remover (TOYOBO Co., Ltd., Osaka, Japan) according to the manufacturer’s instructions. Briefly, 6 μL of RNA at a concentration of 40 ng/μL was mixed with 2 μL of 4 × DN Master Mix containing DNase, corresponding to a total RNA amount of 240 ng, and incubated at 37 °C for 5 min to remove genomic DNA. Subsequently, 2 μL of 5 × RT Master Mix II was added, and reverse transcription was performed at 37 °C for 15 min, followed by 50 °C for 5 min and 98 °C for 5 min, with a final hold at 4 °C.

qRT-PCR was performed using GeneAce SYBR™ qPCR Mix (Nippon Gene, CO., Ltd., Tokyo, Japan) on a Stratagene Mx3005P system (Agilent Technologies, Inc. CA, United States). Relative gene expression levels were normalized to β-actin and calculated using the ΔΔCt method. The primer sequences are listed in Table 2.

**Table 2:** Primer sequences.

Gene	Forward primer (5′ → 3′)	Reverse primer (5′ → 3′)
*HO-1* *	AGCTCTCAAAGCCTCCAACC	TGGTATCTCCTCGCTGGTTC
*p62 (SQSTM1)*	CTGTACGGGCCAGTTTCTCTG	TGTCACTTGTTTTGCTGCCC
*β-Actin*	AACCTAACTTGCGCAGAAAACAAG	TGCTGTCACCTTCACCGTTC

*Heme Oxygenase 1.

### 2.6 Immunofluorescence analysis of Nrf2 nuclear translocation

Cells were cultured in chamber slides and treated with SLE (100 μg/mL) and/or rotenone (200 nM) for 3 h. Cells were fixed with methanol at −20 °C for 5 min, permeabilized with 0.1% Triton X-100, and blocked with serum-free protein block (X0909, Dako, Agilent Technologies) which was 0.25% casein in PBS, containing stabilizing protein and 0.015 mol/L sodium azide.

Cells were incubated overnight at 4 °C with anti-Nrf2 antibody (1:40; Santa Cruz Biotechnology, Inc., TX, United States, sc-365949), followed by Alexa Fluor 488-conjugated secondary antibody (1:200; Thermo Fisher Scientific, Inc., MA, United States, A-11001) for 1 h. Nuclei were stained with DAPI. Fluorescence images were captured using an FSX100 microscope, and nuclear fluorescence intensity was quantified using ImageJ. The true fluorescence intensity of the cells was defined as the value obtained by subtracting the average background fluorescence intensity (measured at 10 locations in the image) from the measured fluorescence intensity of the cell. Graphs were generated based on the 40 cells per group.

### 2.7 Western blot analysis

Cells were lysed in RIPA buffer (Table 3) and centrifuged at 15,000 rpm for 10 min at 4 °C. Protein concentration was determined using a BCA assay (Thermo Fisher Scientific). Equal amounts of protein (20 μg) were separated by SDS-PAGE using 10% gel. Electrophoresis was performed at 90 V and 30 mA through the stacking gel, followed by 160 V and 60 mA through the resolving gel. Proteins were transferred onto polyvinylidene difluoride membranes (IPFL00010; Merck Millipore, Billerica, MA, United States) at 24 V and 150 mA for 20 min.

**Table 3:** Composition of RIPA buffer.

Components	Addition amount (μL)	Final concentration
RIPA stock solution	7,864	
Leupeptin (10 mg/mL DMSO)	8	10 μg/mL
Pepstatin (10 mg/mL DMSO)	8	10 μg/mL
NaF (1 M)	40	5 mM
Na_3_VO_4_(200 mM)	40	1 mM
PMSF (200 mM)	40	1 mM

RIPA Stock solution containing 1% NP-40, 150 mM NaCl, 0.5% Sodium deoxycholate, 0.1% Sodium dodecyl sulfate, 1 mM EDTA·2Na and 50 mM Tris–HCl (pH7.4).

Membranes were blocked with 3% bovine serum albumin in Tris-Buffered saline containing 0.1% Tween 20 (TBST) for 1 h room temperature, and then incubated overnight at 4 °C with the following primary antibodies: AMPKα rabbit monoclonal antibody (1:1,000; Cell Signaling Technology, Inc., MA, United States, #2532), phospho-AMPKα (Thr172) rabbit monoclonal antibody (1:1,000; Cell Signaling Technology, Inc., #2531), Parkin antibody (1:500; Cell Signaling Technology, Inc., #2132), PINK1 polyclonal antibody (1:1,000; Proteintech·Japan, Tokyo, Japan, 23,274-1-AP), LC3A/B (D3U4C) XP^®^ rabbit monoclonal antibody (1:1,000; Cell Signaling Technology, Inc., #12741), p62/SQSTM1 polyclonal antibody (1:10,000; Proteintech·Japan, 18,420-1-AP), phospho-p62/SQSTM1 (Ser349) polyclonal antibody (1:5,000; Proteintech·Japan, 29,503-1-AP), and GAPDH (D16H11) rabbit monoclonal antibody (1:10,000; Cell Signaling Technology, Inc., #5174). After incubation with HRP-conjugated secondary antibodies for 1 h, protein bands were visualized using chemiluminescence (EZ West Lumi Plus, 2,332,638, ATTO) and quantified using CS Analyzer 3 software (ATTO). The band quantification was normalized exclusively to GAPDH. Western blot analyses were performed in three independent experiments. The exposure times have been added to the respective membrane photographs in Supplementary material.

### 2.8 Measurement of mitochondrial membrane potential

Mitochondrial membrane potential was assessed using the Multi-Parameter Apoptosis Assay Kit (Cayman Chemical). Cells were treated with SLE (100 μg/mL) and/or rotenone (200 nM) for 24 h. The cells were then incubated for 15 min with a staining solution containing TMRE-based solution including tetramethylrhodamine ethyl ester (TMRE) dye, Hoechst Dye and RedDot™2 Viability Dye prepared in PBS for 15 min at 37 °C according to the manufacturer’s instructions. The TMRE-based solution was then removed, and the cells were washed with PBS. Subsequently, 100 μL of PBS was added, 24 × 24 mm coverslip was placed over the cells. Fluorescence image was acquired using a fluorescence microscope, and TMRE fluorescence intensity was quantified using ImageJ software. The true fluorescence intensity of the cells was defined as the value obtained by subtracting the average background fluorescence intensity (measured at 10 locations in the image) from the measured fluorescence intensity of the cell. Graphs were generated based on the 40 cells per group. TMRE fluorescence intensity was compared among groups under identical staining and imaging conditions; however, a positive control for mitochondrial membrane depolarization was not included in this experiment.

### 2.9 Assessment of nuclear morphology

Cells were treated with SLE (100 μg/mL) and/or rotenone (200 nM) for 24 h. The cells were fixed with 70% ethanol for 30 min at 4 °C. After removing of the ethanol, the cells were stained with 50 μg/mL propidium iodide (PI) in PBS containing 0.1% Triton-X and 100 μg/mL RNase for 1 h at 37 °C. The staining solution was then removed, and the cells were washed with PBS. Subsequently, 300 μL of PBS was added, 24 × 24 mm coverslip was placed over the cells. Nuclear morphology was observed using a fluorescence microscope (Olympus LSC101). Four images were captured per group, with approximately 150 cells per image, and the results were evaluated qualitatively. Images were captured by an investigator who was blinded to the group allocation.

### 2.10 Animal experiments

All animal experiments were approved by the Institutional Animal Care and Use Committee of Osaka Metropolitan University (approval number: S0144). Twenty-eight Male C57BL/6 J mice (6 weeks old) were acclimated for 4 days and then randomly assigned to four groups: control, rotenone, low-dose SLE (0.07%), and high-dose SLE (0.13%), based on body weight and baseline motor function test results. Rotenone was orally administered at a dose of 10 mg/kg body weight once daily for 35 days. The composition of the experimental diets is shown in Table 4. The sample size was determined based on previous studies and preliminary experiments in which significant differences were detected with a similar number of animals. To account for expected variability, seven mice were assigned to each group. No animals or data points were excluded from the analysis.

**Table 4:** Composition of experimental diets.

Components (g)	Control	0.07% SLE	0.13% SLE
Casein	140	140	140
L-cystine	1.8	1.8	1.8
Cornstarch	465.692	464.992	464.392
α-cornstarch	155	155	155
Sucrose	100	100	100
Soybean oil	40	40	40
Cellulose powder	50	50	50
AIN-93 M mineral	35	35	35
AIN-93 vitamin	10	10	10
Choline hydrogen tartrate	2.5	2.5	2.5
tert-Butylhydroquinone	0.008	0.008	0.008
Strawberry leaf extract	0	0.7	1.3
Total	1,000	1,000	1,000

SLE was incorporated into the diet at concentrations of 0.07 and 0.13%. These concentrations were selected empirically before the start of the experiment based on the effective concentration observed in the *in vitro* experiments (100 μg/mL), the approximate body weight and expected daily food intake of the mice, and the limited intestinal absorption generally reported for dietary polyphenols. Based on these considerations, an approximate dietary concentration range was estimated and 0.07 and 0.13% were selected as practical low- and high-dose concentrations, respectively. This estimation was used only as a guide for dose selection and was not intended to predict the actual circulating concentrations of SLE or its individual constituents. Food intake was monitored throughout the experimental period to estimate the actual intake of SLE. The average daily food intake per mouse was 4.89 g in the low-dose SLE group and 5.23 g in the high-dose SLE group. Based on these values, the estimated daily intake of SLE was approximately 3.42 mg/day/mouse in the low-dose group and 6.80 mg/day/mouse in the high-dose group.

Mice were maintained under controlled conditions (25 °C, 12 h light/dark cycle) with free access to food and water. To minimize potential confounding factors, animals were randomly assigned to cages and treatment groups. Measurements and sample collection were performed under the same experimental conditions and at approximately the same time of day.

At the end of the experiment, mice were deeply anesthetized with 5% isoflurane inhalation. Adequate anesthesia was confirmed by the loss of righting reflex and pain response. Whole blood samples were collected via the posterior vena cava, and after confirmation of death, brain tissues were collected.

### 2.11 Motor function tests

#### 2.11.1 Rotarod test

Motor coordination and balance were assessed using a rotarod apparatus (Rota-rod Treadmill 370, BIO MEDICA) equipped with a rod 6 cm in diameter. Before the experimental measurements, the mice underwent one preliminary training session to become familiar with the apparatus. During each test, the rotation speed was linearly accelerated from 6 to 25 rpm over 5 min, corresponding to an acceleration rate of approximately 3.8 rpm/min. The maximum trial duration was 300 s. Each mouse underwent three trials per test session, and rotarod testing was performed five times at 1-week intervals. The latency to fall was recorded, and the mean value of the three trials was used as the representative value for each mouse.

#### 2.11.2 Pole test

Motor coordination and bradykinesia were assessed using the pole test. The apparatus consisted of a vertical iron pole measuring 75 cm in height and 0.8 cm in diameter. Each mouse was placed head-upward at the top of the pole. The mouse was then allowed to turn downward and descend the pole voluntarily. The time required for the mouse to turn completely from the initial head-upward position to a head-downward position was recorded as the T-turn time. The total descent time was defined as the time elapsed from placement of the mouse at the top of the pole until the mouse completed the T-turn and reached the ground with all four paws. Each mouse underwent three trials per test session, and the mean value of the three trials was used for statistical analysis. No maximum cut-off time was imposed, and the time required for each mouse to complete the descent was recorded.

#### 2.11.3 Wire hang test

Forelimb grip strength and motor coordination were assessed using the wire-hang test. The apparatus consisted of a square wire grid measuring 30 × 30 cm, with a mesh size of 1 × 1 cm. The grid was positioned 35 cm above a soft padded surface to prevent injury in the event of a fall. Each mouse was placed on the grid and allowed to grasp the wire mesh. The grid was then gently inverted, and the latency to fall was recorded. Each mouse underwent three trials per test session, with a maximum cut-off time of 300 s for each trial. Mice that remained suspended for 300 s were assigned a value of 300 s. The mean latency from the three trials was used for statistical analysis.

Because there were no substantial differences in body weight among the experimental groups, hanging performance was not adjusted for body weight.

### 2.12 Immunohistochemistry for tyrosine hydroxylase

Brain tissues were fixed in a 10% neutral buffered formalin. The fixed brain samples were sent to the Biopathology Institute, Inc. (Oita, Japan), for pathological and immunohistochemical specimen preparation within 2 days after sacrifice.

Immunohistochemical staining for TH was performed using the labeled streptavidin-biotin method. The procedure was carried out in accordance with our previous study (24). Briefly, specimens were treated with 3% hydrogen peroxide for 5 min to block endogenous peroxidase activity and then Serum-free Protein Block (X0909, Dako, Agilent Technologies) for 5 min. The sections were subsequently incubated with an anti-TH antibody (1:80, Sigma-Aldrich Co. LLC, MO, United States, AB152) for 1 h at room temperature. They were then incubated with polyclonal goat anti-rabbit immunoglobulins/Biotinylated (1:400, Dako, Agilent Technologies, E0432), followed by Streptavidin/HRP (1:400, Dako, Agilent Technologies, P0397) for 30 min at room temperature. They were rinsed in 0.01 M PBS, pH 7.2, after each incubation step. The peroxidase reaction was visualized by the use of 3,3′-diaminobenzidine tetrahydrochloride (0.2 mg/mL) with nickel chloride color modification.

Images were captured using a microscope (Olympus IX-70). The staining intensity in the substantia nigra regions of the left and right hemispheres was quantified using ImageJ. The corrected staining intensity was calculated by subtracting the average background intensity, measured at 40 locations in each image, from the measured staining intensity of the TH-positive area. Graphs were generated based on measurements from 40 cells per group. Images and analysis were performed by an investigator who was blinded to the group allocation.

### 2.13 Statistical analysis

Data are presented as mean ± SD, except for the motor function test data from the *in vivo* experiments, which are presented as the mean ±SE. Statistical analysis was performed using one-way ANOVA, followed by Tukey–Kramer multiple comparison test or Dunnett’s multiple-comparison test, as appropriate. Dunnett’s test was used for the motor function data from the *in vivo* experiments. Differences were considered statistically significant at *p* < 0.05.

## 3 Results

### 3.1 Effects of rotenone and SLE on SH-SY5Y cell viability

**Figure 1:**
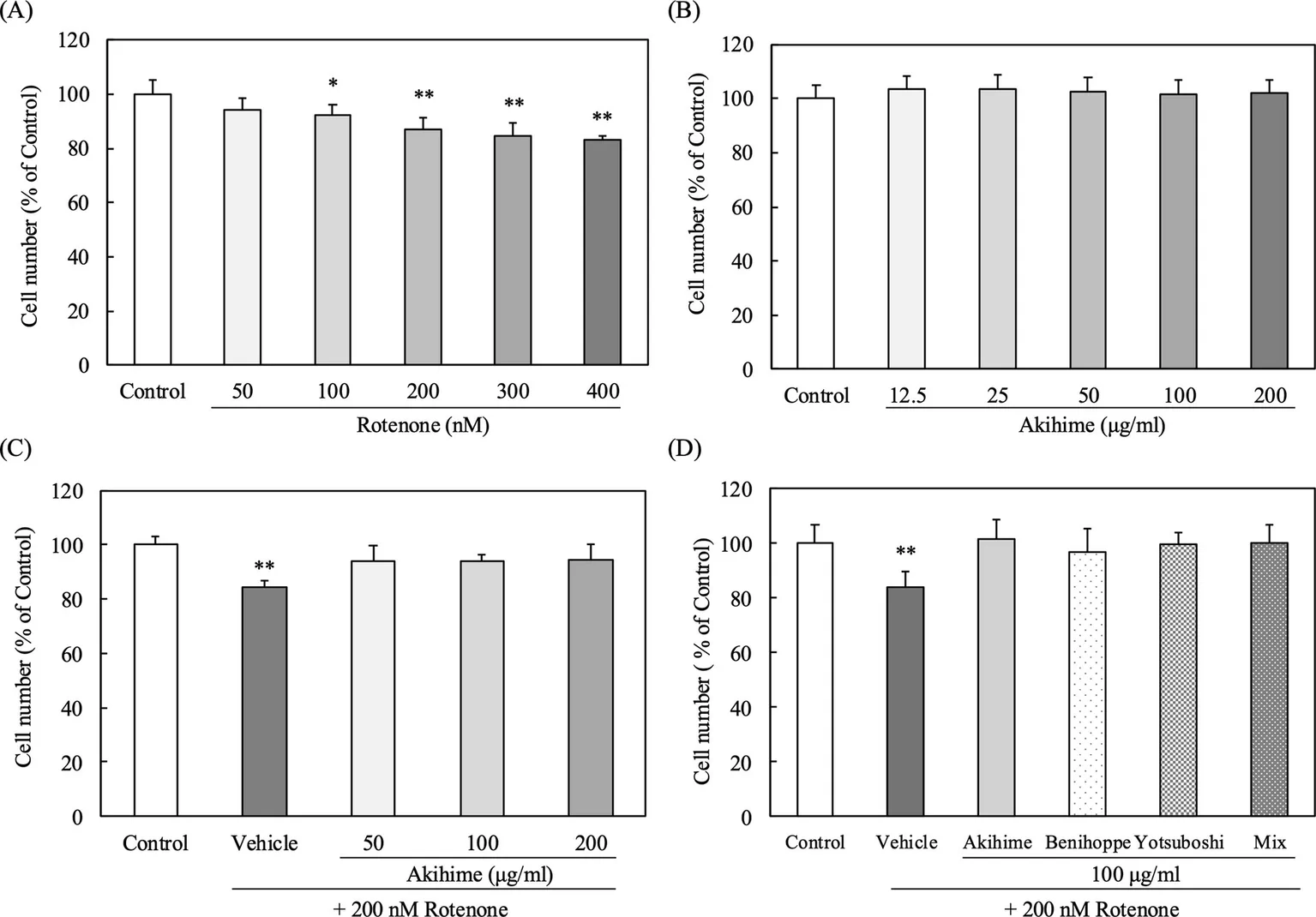
Effects of rotenone and SLE on SH-SY5Y cell viability. **(A)** SH-SY5Y cells were treated with increasing concentrations of rotenone (0–400 nM) for 24 h, and cell viability was assessed by the MTT assay. **(B)** Cells were treated with SLE (Akihime) (0–200 μg/mL) for 24 h, and cell viability was evaluated by the MTT assay. **(C)** Cells were co-treated with 200 nM rotenone and SLE (0–200 μg/mL) for 24 h. **(D)** Cells were co-treated with 200 nM rotenone and SLE prepared from different strawberry cultivars (Akihime, Benihoppe, Yotsuboshi, and their mixture; 100 μg/mL) for 24 h. Data are presented as mean ± SD (*n* = 6). *p < 0.05, **p < 0.01 vs. control.

The effects of rotenone and strawberry leaf extract (SLE) on SH-SY5Y cell viability were evaluated using the MTT assay after 24 h of treatment. To determine an appropriate rotenone concentration for the *in vitro* PD model, cells were exposed to 0–400 nM rotenone. Rotenone significantly decreased cell viability in a concentration-dependent manner (Figure 1A). Based on these results, 200 nM rotenone was selected for subsequent experiments.

Next, the effect of SLE (Akihime) alone on SH-SY5Y cells was examined. SLE did not affect cell viability at concentrations ranging from 0 to 200 μg/mL (Figure 1B), indicating no apparent cytotoxicity under the present experimental conditions.

To evaluate its protective effect, cells were co-treated with 200 nM rotenone and various concentrations of SLE. Rotenone-induced reduction in cell viability was significantly attenuated by SLE at 50 and 100 μg/mL (Figure 1C). We then compared extracts prepared from different strawberry cultivars, including Akihime, Benihoppe, Yotsuboshi, and a mixture of the three cultivars, at 100 μg/mL. All extracts showed comparable protective effects against rotenone-induced loss of viability, with no significant differences among cultivars (Figure 1D). Akihime showed the highest total polyphenol content among the tested cultivars and was selected as a representative SLE preparation for subsequent *in vitro* experiments. However, total polyphenol content does not provide information regarding the qualitative or quantitative composition of individual phenolic constituents, and therefore the phytochemical basis for the biological activity observed among the different cultivars remains to be determined.

### 3.2 Effects of rotenone and SLE on intracellular ROS production

**Figure 2:**
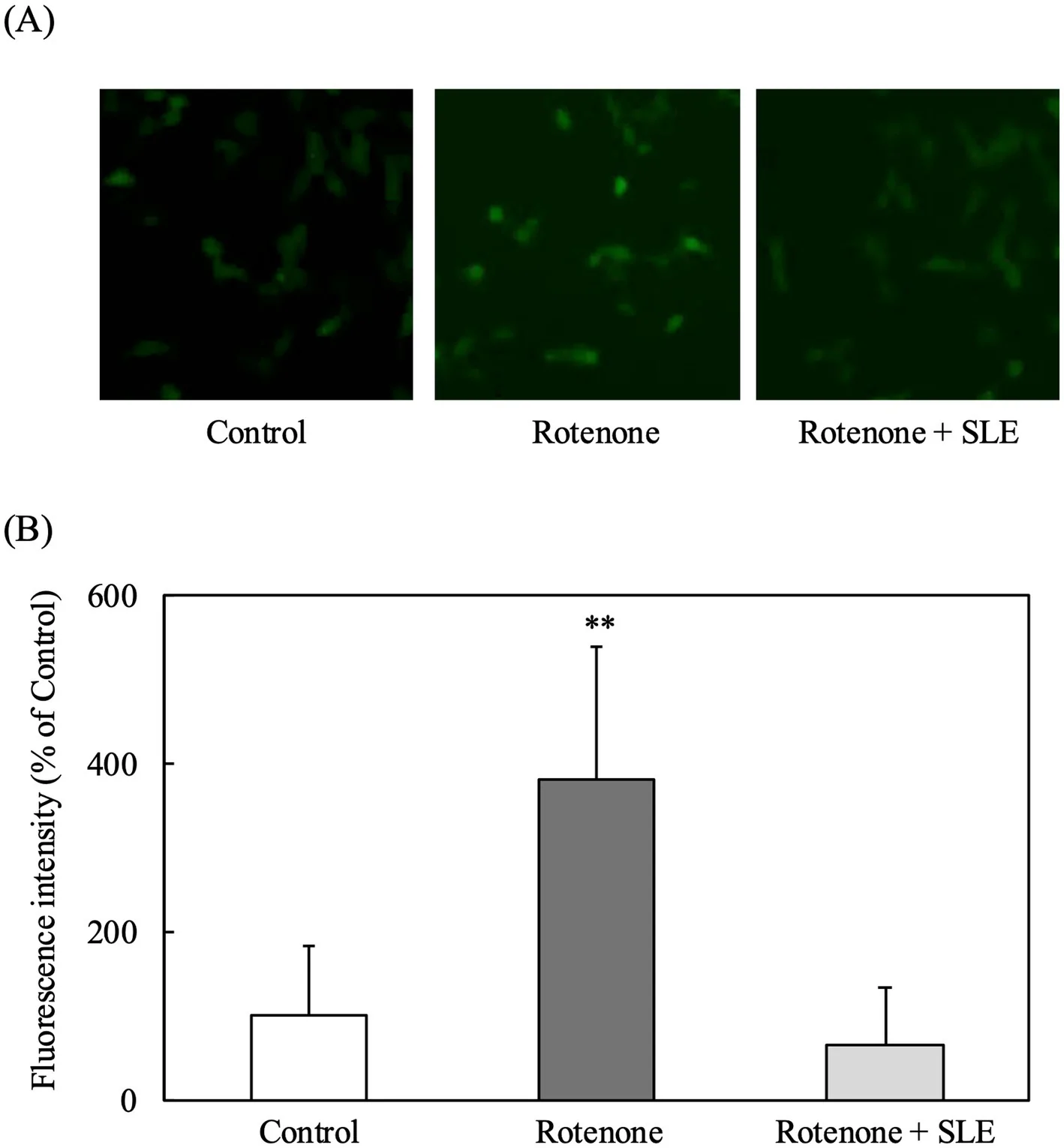
Effects of rotenone and SLE on intracellular ROS production. SH-SY5Y cells were treated with 200 nM rotenone in the presence or absence of SLE (100 μg/mL) for 6 h. Intracellular ROS levels were assessed using DCFH-DA staining. **(A)** Representative fluorescence images showing intracellular ROS levels. **(B)** Quantification of fluorescence intensity. Data are presented as mean ± SD from measurements of 40 cells. **p < 0.01 vs. control.

To assess intracellular ROS production, SH-SY5Y cells were stained with DCFH-DA after 6 h of treatment. Rotenone treatment markedly increased intracellular ROS levels, as indicated by enhanced green fluorescence compared with the control group (Figures 2A,B). Co-treatment with SLE markedly reduced rotenone-induced fluorescence intensity.

### 3.3 Effect of SLE on HO-1 mRNA expression

**Figure 3:**
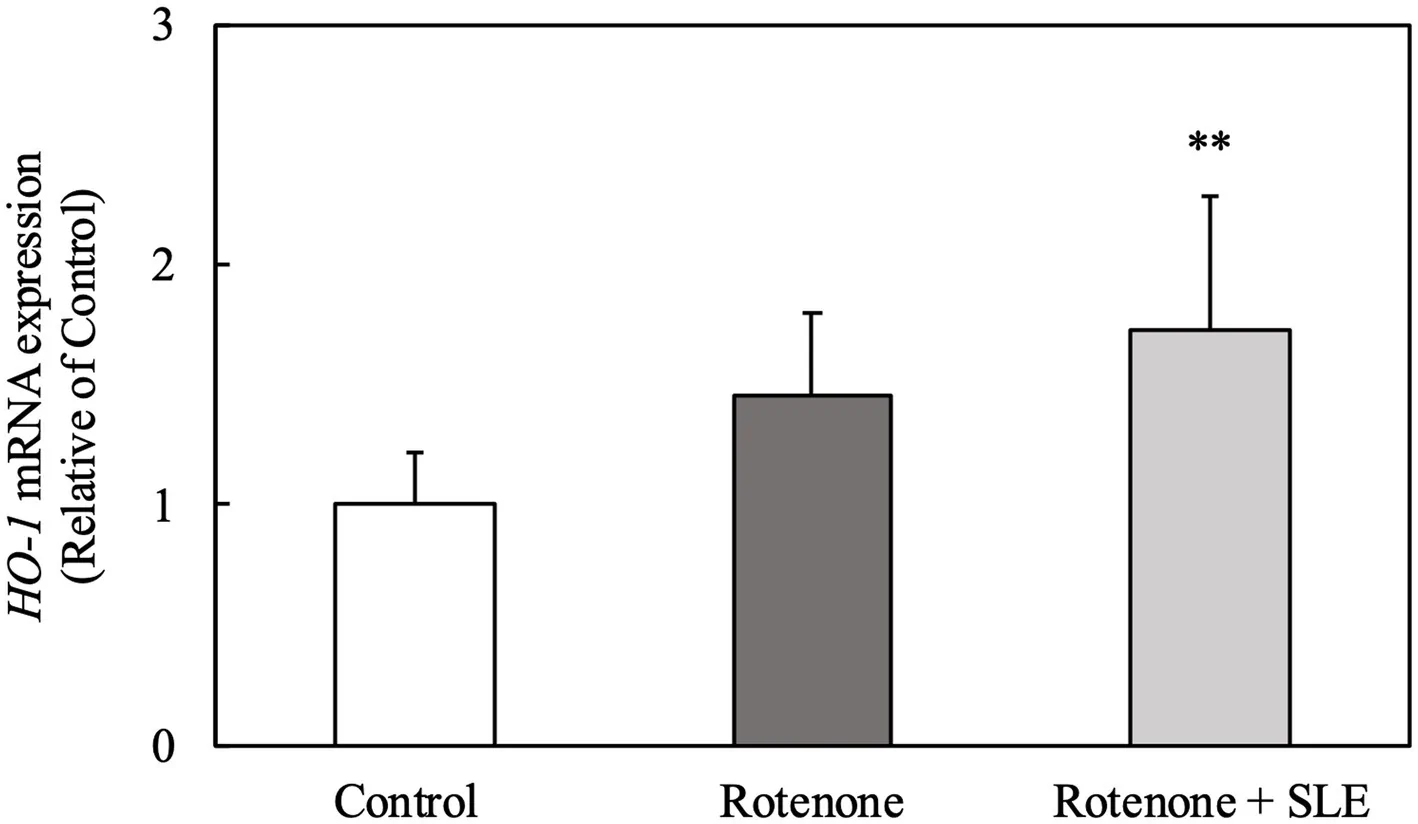
Effect of SLE on HO-1 mRNA expression. SH-SY5Y cells were treated with 200 nM rotenone in the presence or absence of SLE (100 μg/mL) for 6 h. HO-1 mRNA expression was quantified by real-time PCR. Data are presented as mean ± SD (*n* = 4). **p < 0.01 vs. control.

To investigate the mechanism underlying the suppression of ROS production by SLE, we examined the mRNA expression of heme oxygenase-1 (HO-1), a representative antioxidant enzyme regulated by the Nrf2 pathway (25). HO-1 mRNA expression was significantly higher in the SLE-treated group than in the rotenone-alone group after 6 h of treatment (Figure 3).

### 3.4 Effects of rotenone and SLE on nuclear translocation of Nrf2

**Figure 4:**
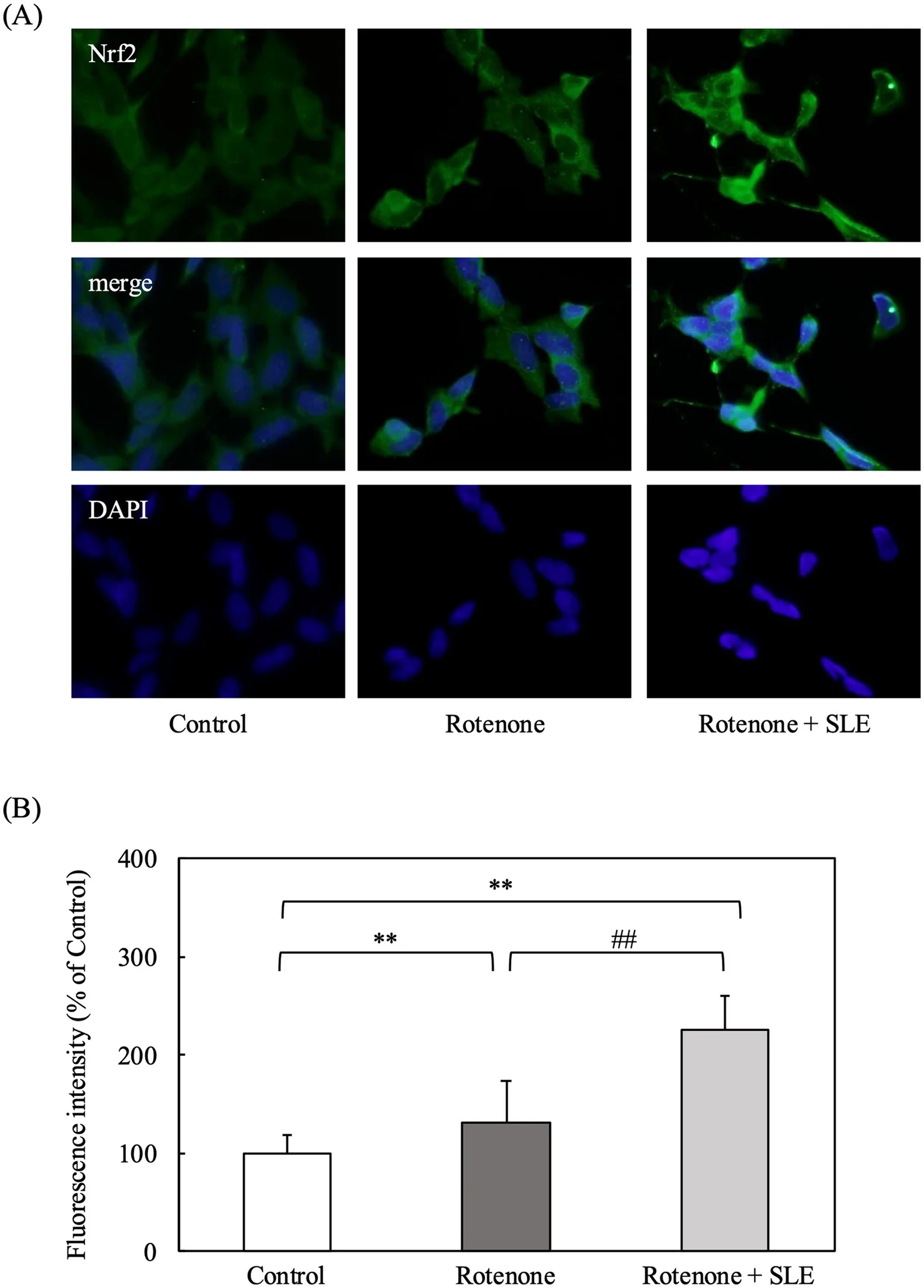
Effects of rotenone and SLE on nuclear translocation of Nrf2. SH-SY5Y cells were treated with 200 nM rotenone in the presence or absence of SLE (100 μg/mL) for 3 h. Cells were subjected to immunofluorescence staining for Nrf2, and nuclei were counterstained with DAPI. **(A)** Representative images showing Nrf2 localization (green) and nuclei (blue). **(B)** Quantification of nuclear Nrf2 fluorescence intensity. Data are presented as mean ± SD from measurements of 40 cells. **p < 0.01 vs. control; ##*p* < 0.01 vs. rotenone-treated group.

Because SLE increased HO-1 expression, we next investigated the nuclear translocation of Nrf2, a key transcription factor regulating antioxidant gene expression. Immunofluorescence staining revealed minimal nuclear localization of Nrf2 in the control group, whereas a slight increase was observed in the rotenone-treated group (Figures 4A,B). Notably, co-treatment with SLE and rotenone significantly increased nuclear Nrf2 fluorescence compared with rotenone treatment alone.

### 3.5 Involvement of AMPK in SLE-induced nuclear translocation of Nrf2

**Figure 5:**
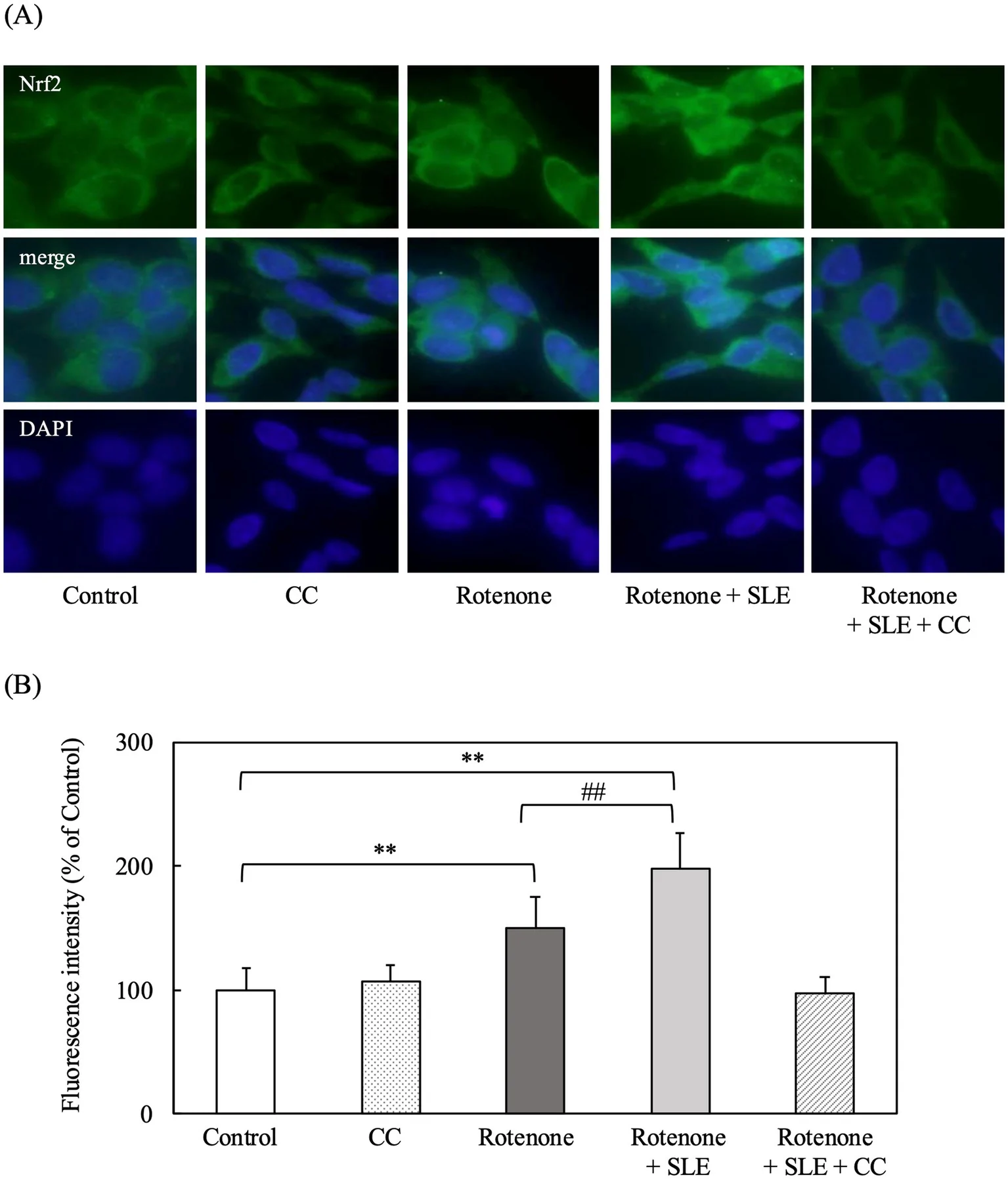
Involvement of AMPK in SLE-induced nuclear translocation of Nrf2. SH-SY5Y cells were treated with Compound C (4 μM), rotenone (200 nM), and/or SLE (100 μg/mL) for 3 h. Nrf2 localization was evaluated by immunofluorescence staining. **(A)** Representative images showing Nrf2 (green) and nuclei (blue). **(B)** Quantification of nuclear Nrf2 fluorescence intensity. Data are presented as mean ± SD from measurements of 20 cells. **p < 0.01 vs. control; ##*p* < 0.01 vs. rotenone-treated group.

To examine whether AMPK is involved in SLE-induced Nrf2 nuclear translocation (26), SH-SY5Y cells were treated with Compound C (CC), an AMPK inhibitor, in the presence or absence of SLE and rotenone. Nuclear Nrf2 fluorescence was low in the control and CC-alone groups, and slightly increased in the rotenone-treated group (Figures 5A,B). Co-treatment with SLE and rotenone markedly increased nuclear Nrf2 localization, whereas this increase was attenuated by CC treatment.

### 3.6 Effect of SLE on AMPK phosphorylation

**Figure 6:**
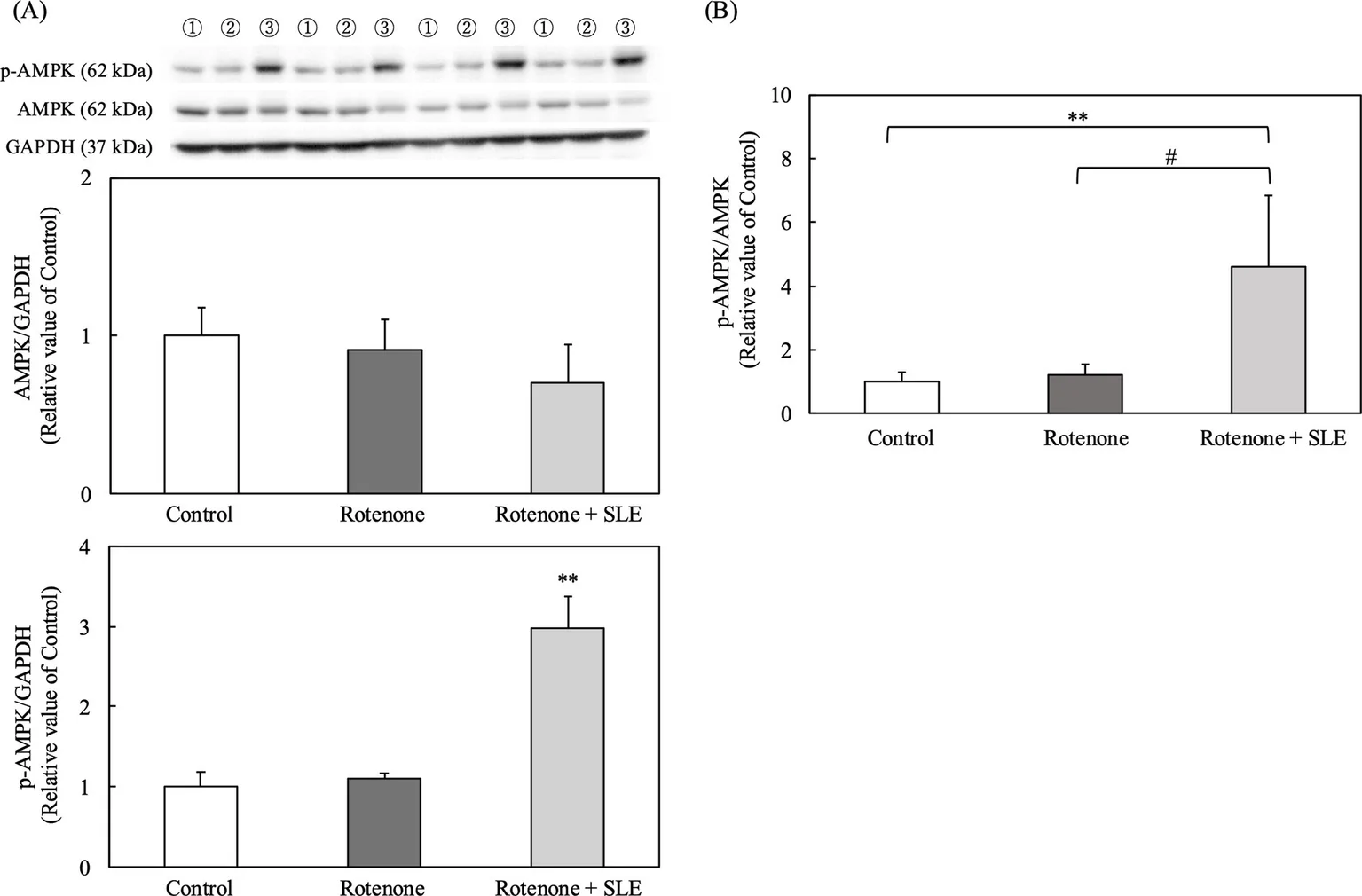
Effect of SLE on AMPK phosphorylation. SH-SY5Y cells were treated with 200 nM rotenone in the presence or absence of SLE (100 μg/mL) for 2 h. Protein levels of phosphorylated AMPK (p-AMPK) and total AMPK were analyzed by Western blotting. The p-AMPK/AMPK ratio was quantified and normalized to the control group. Data are presented as mean ± SD (*n* = 4). **p < 0.01 vs. control; #*p* < 0.05 vs. rotenone-treated group.

To further evaluate AMPK activation, the protein levels of phosphorylated AMPK (p-AMPK) and total AMPK were analyzed by Western blotting after 2 h of treatment. SLE increased the p-AMPK/AMPK ratio compared with the rotenone-treated group (Figure 6).

### 3.7 Effects of rotenone and SLE on p62 mRNA expression

**Figure 7:**
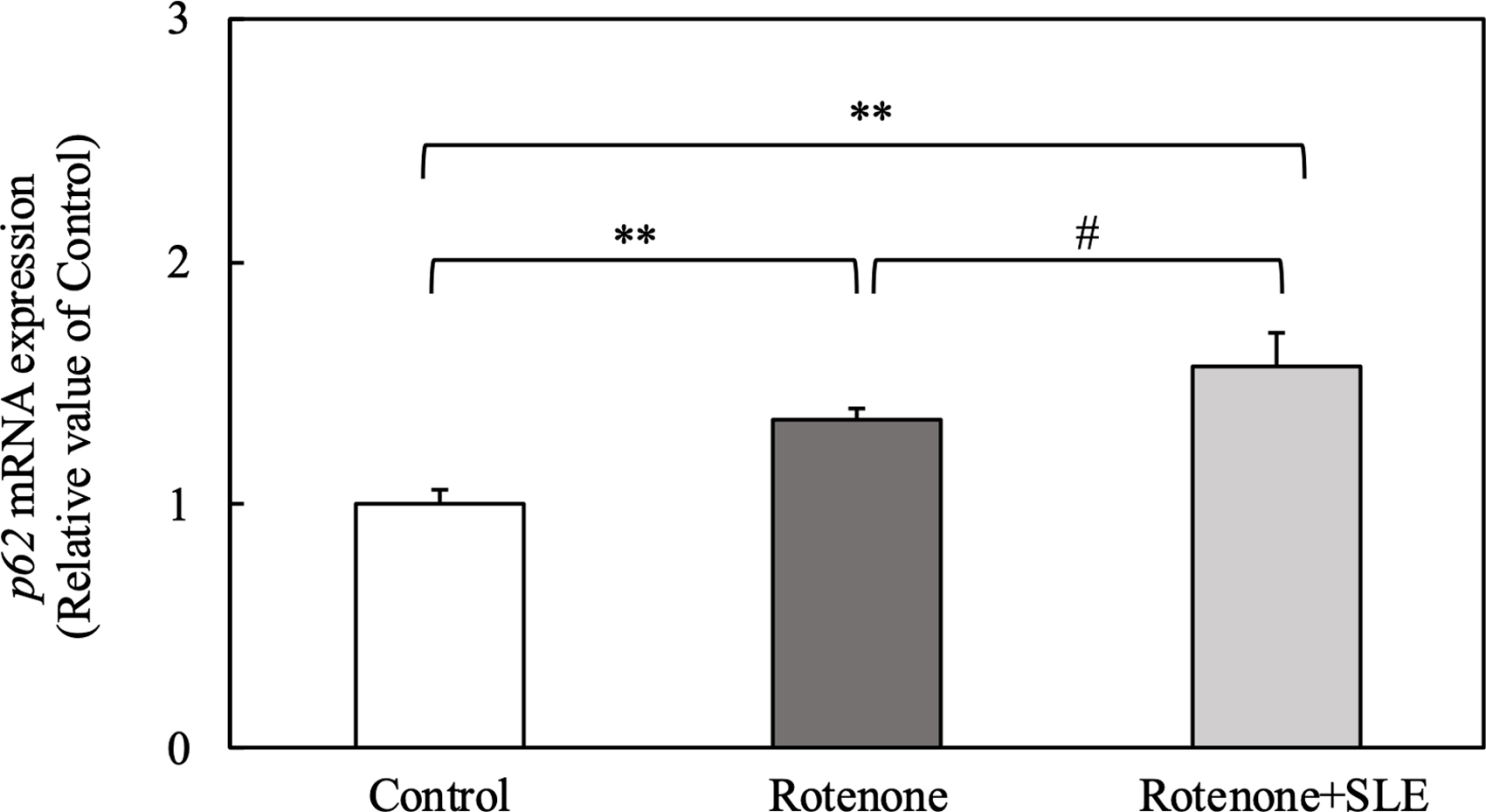
Effects of rotenone and SLE on *p62* mRNA expression. SH-SY5Y cells were treated with 200 nM rotenone in the presence or absence of SLE (100 μg/mL) for 6 h. *p62* mRNA expression was quantified by real-time PCR. Data are presented as mean ± SD (*n* = 3). **p < 0.01 vs. control; #*p* < 0.05 vs. rotenone-treated group.

Because p62 is a downstream target associated with Nrf2 signaling, we next examined p62 mRNA expression after 6 h of treatment. p62 mRNA levels were significantly increased in the SLE-treated group compared with the rotenone-alone group (Figure 7).

### 3.8 Effects of rotenone and SLE on mitophagy-related pathways

To investigate whether SLE affects mitochondrial quality control pathways, we analyzed several mitophagy-related molecules and mitochondrial membrane potential in rotenone-treated SH-SY5Y cells.

**Figure 8:**
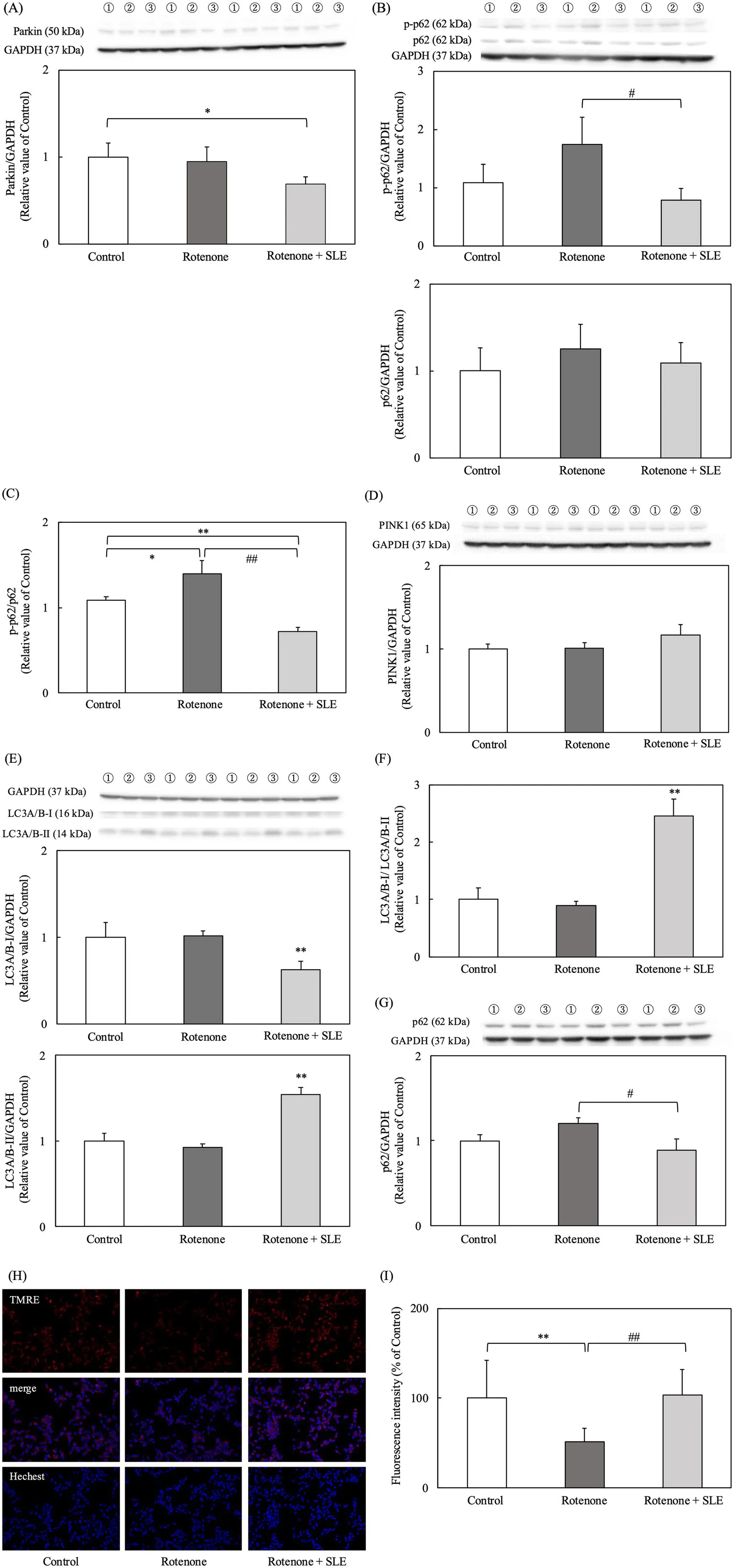
Effects of rotenone and SLE on mitophagy-related pathways. SH-SY5Y cells were treated with 200 nM rotenone in the presence or absence of SLE (100 μg/mL). **(A)** Parkin protein levels after 6 h of treatment. **(B)** Phosphorylated p62 (p-p62), total p62, and **(C)** the p-p62/p62 ratio after 6 h of treatment. **(D)** PINK1 protein levels after 6 h of treatment. **(E)** LC3-I, LC3-II, and **(F)** LC3-II/LC3-I ratio after 24 h of treatment. **(G)** p62 protein levels after 24 h of treatment. **(H)** Representative images of mitochondrial membrane potential assessed by TMRE staining after 24 h of treatment. **(I)** Quantification of TMRE fluorescence intensity. Data are presented as mean ± SD (*n* = 3–4 for Western blotting; *n* = 40 cells for fluorescence analysis). *p < 0.05, **p < 0.01 vs. control; #*p* < 0.05, ##*p* < 0.01 vs. rotenone-treated group.

After 6 h of treatment, Parkin protein levels were significantly decreased in the SLE-treated group compared with the rotenone-alone group (Figure 8A). In addition, the ratio of phosphorylated p62 to total p62 was also reduced by SLE treatment (Figures 8B,C). Under the same conditions, a slight increase in PINK1 protein expression was observed (Figure 8D).

After 24 h of treatment, the LC3-II/LC3-I ratio increased and p62 protein levels decreased in the SLE-treated group (Figures 8E–G). In parallel, rotenone-induced reduction in mitochondrial membrane potential, assessed by TMRE staining, was alleviated by SLE treatment (Figures 8H,I).

### 3.9 Effect of SLE on rotenone-induced nuclear morphological changes associated with cell death

**Figure 9:**
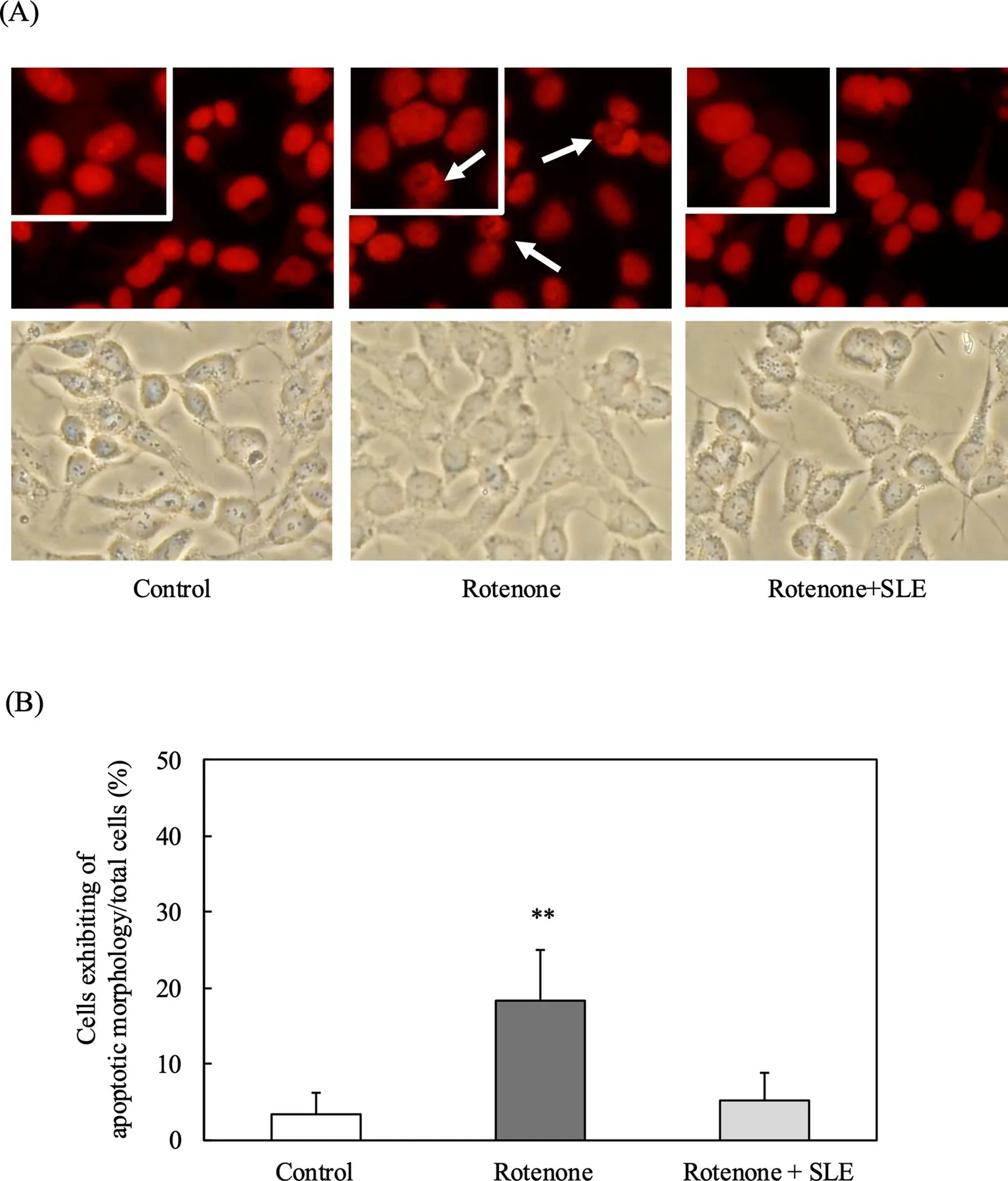
Effect of SLE on rotenone-induced nuclear morphological changes. SH-SY5Y cells were treated with 200 nM rotenone in the presence or absence of SLE (100 μg/mL) for 24 h. **(A)** Nuclear morphology was assessed by propidium iodide (PI) staining. Representative images show nuclear condensation and morphological changes associated with apoptotic cell death. **(B)** Quantification of cells exhibiting of apoptotic morphology/total cells. Data are presented as mean ± SD from measurements of 500 ~ 660 cells. **p < 0.01 vs. control.

To assess the effect of SLE on rotenone-induced cell death, nuclear morphology was examined by PI staining after 24 h of treatment. In the rotenone-treated group, nuclear condensation and other abnormal nuclear morphological changes were observed (Figure 9A). In contrast, these changes were significantly attenuated in cells co-treated with SLE (Figure 9B).

### 3.10 Effects of rotenone and SLE on motor dysfunction in mice

**Figure 10:**
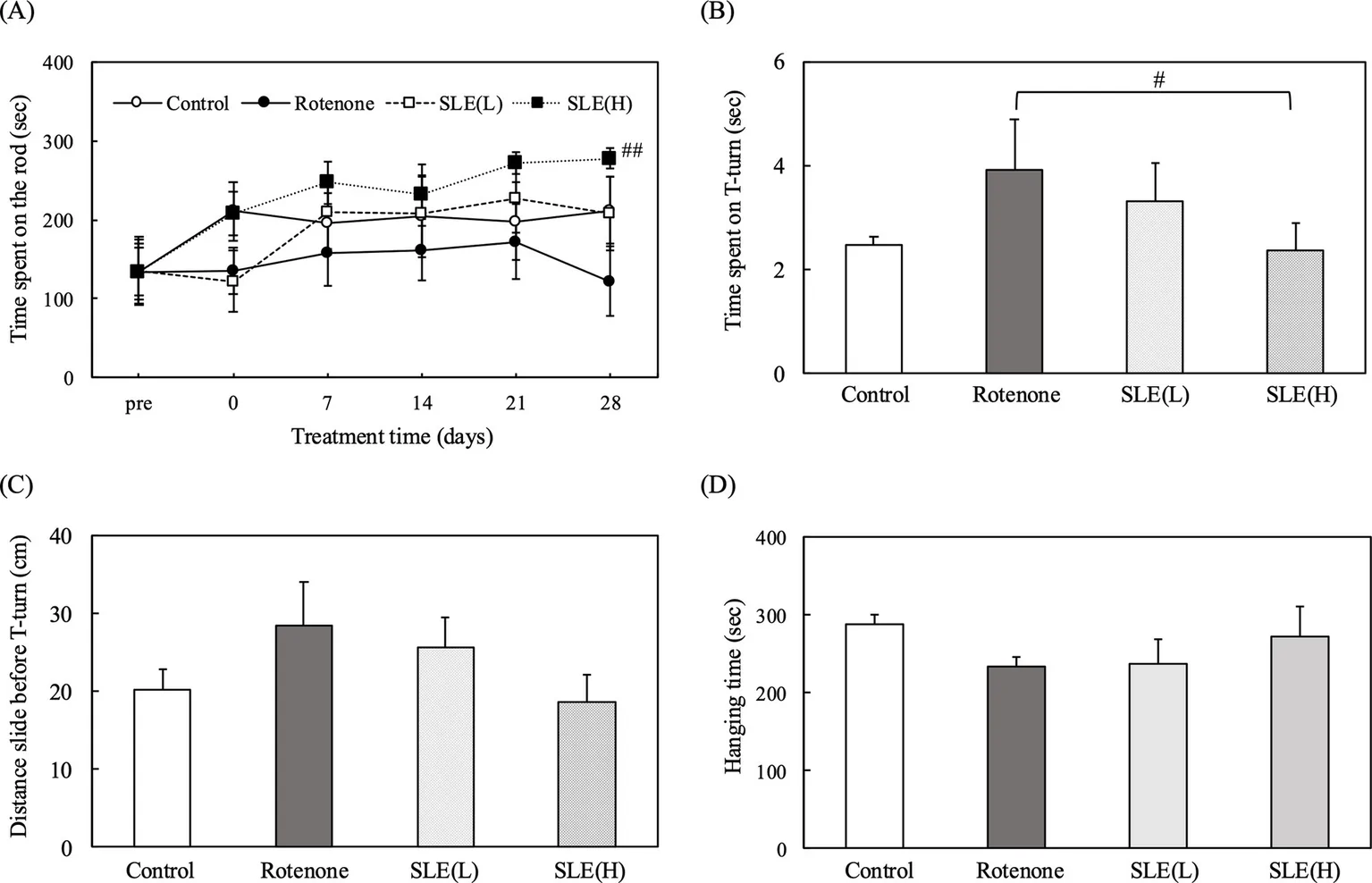
Effects of rotenone and SLE on motor function in mice. Motor function was evaluated using the rotarod, pole test, and wire hang test. **(A)** Rotarod test (latency to fall). **(B)** T-turn time in the pole test. **(C)** Total time in the pole test. **(D)** Wire hang test (hanging time). Data are presented as mean ± SE. #*p* < 0.05, ##*p* < 0.01 vs. rotenone-treated group.

To evaluate the protective effect of SLE *in vivo*, motor function was assessed in rotenone-treated mice using the rotarod test, pole test, and wire hang test. In the rotarod test, rotenone treatment shortened the latency to fall, indicating impaired motor coordination, whereas SLE administration improved performance. A significant difference was observed between the rotenone group and the high-dose SLE group on day 28 (Figure 10A).

In the pole test, rotenone treatment increased both T-turn time and the distance slid before turning, indicating bradykinesia-related impairment (Figures 10B,C). These changes were attenuated in the SLE-treated groups. In particular, the T-turn time was significantly shorter in the SLE(H) group.

In the wire hang test, rotenone treatment reduced hanging time, whereas SLE administration showed a tendency to suppress this reduction (Figure 10D).

### 3.11 Effects of rotenone and SLE on TH expression in the substantia nigra

**Figure 11:**
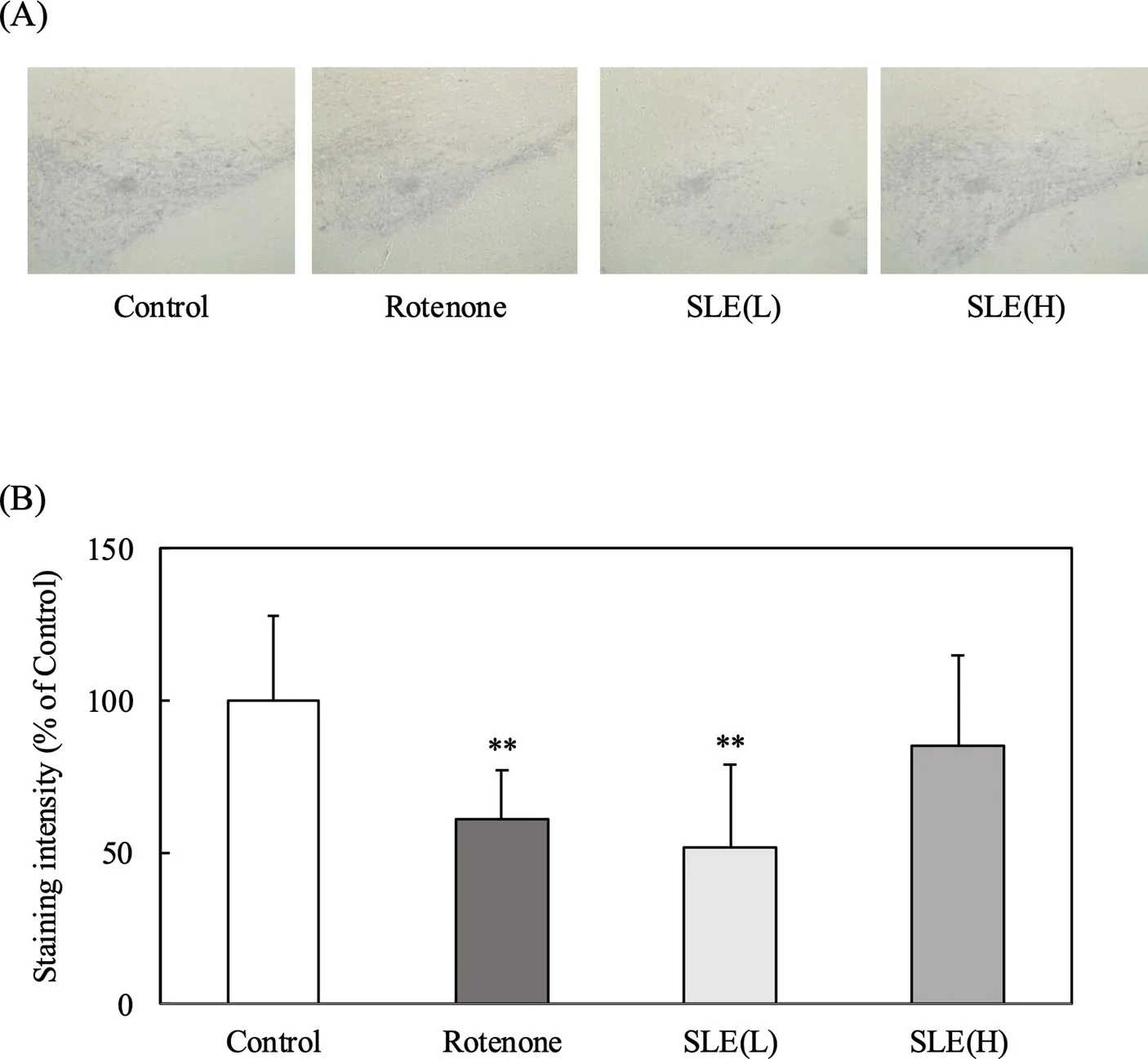
Effects of rotenone and SLE on tyrosine hydroxylase expression in the substantia nigra. **(A)** Representative immunohistochemical images of tyrosine hydroxylase (TH)-positive neurons in the substantia nigra (original magnification ×100). **(B)** Quantification of TH-positive staining intensity. Data are presented as mean ± *SD*. **p < 0.01 vs. control.

To examine whether the behavioral effects of SLE were associated with changes in the nigrostriatal dopaminergic system, TH expression in the substantia nigra was evaluated by immunohistochemical staining. Rotenone treatment significantly reduced TH immunoreactivity in the substantia nigra compared with the control group. SLE administration attenuated the rotenone-induced reduction in TH immunoreactivity, with greater preservation of TH staining observed in the SLE-treated groups (Figures 11A,B).

## 4 Discussion

The present study provides evidence that strawberry leaf extract (SLE) attenuates rotenone-induced dopaminergic neuronal damage *in vitro* and *in vivo*. The protective effects of SLE were associated with reduced ROS accumulation, increased Nrf2 nuclear localization and HO-1 expression, changes in mitochondrial homeostasis-related markers, restoration of mitochondrial membrane potential, and attenuation of cell death-related nuclear morphological changes.

Rotenone is a well-established mitochondrial complex I inhibitor widely used to model Parkinson’s disease (PD) pathology, as it reproduces key features including oxidative stress, mitochondrial dysfunction, and dopaminergic neuronal loss (27, 28). In the present study, rotenone induced a concentration-dependent reduction in SH-SY5Y cell viability, and 200 nM was selected to induce moderate but reproducible neuronal injury. Under these conditions, SLE exhibited no cytotoxicity and significantly attenuated rotenone-induced cell death. SLE exhibited no apparent cytotoxicity and significantly attenuated the rotenone-induced reduction in cell viability. Comparable protective effects were observed among extracts prepared from different strawberry cultivars. However, because only total polyphenol content was determined in the present study, these findings cannot be attributed to specific polyphenolic constituents or to a particular phenolic profile. Extracts with similar total polyphenol contents may differ substantially in their qualitative and quantitative phytochemical composition, including the relative abundance of ellagitannins, flavonols, proanthocyanidins, phenolic acids, and other constituents. Thus, the present findings support the protective activity of SLE as a whole extract, whereas the individual compounds or combinations of compounds responsible for this activity remain to be identified.

Oxidative stress is a central driver of PD pathogenesis and is closely linked to mitochondrial dysfunction and neuronal vulnerability (29, 30). Consistent with previous reports, rotenone markedly increased intracellular ROS levels, whereas SLE effectively suppressed ROS accumulation. Importantly, the magnitude of ROS suppression observed in this study suggests that the neuroprotective effects of SLE cannot be explained solely by direct radical scavenging activity. Instead, these effects are more likely mediated through activation of endogenous antioxidant systems, a concept increasingly recognized as a key mechanism underlying the biological activity of dietary polyphenols (31).

The Nrf2–ARE signaling pathway represents a master regulatory system governing cellular redox homeostasis (31, 32). Here, SLE promoted nuclear translocation of Nrf2 and upregulated HO-1 expression, indicating activation of the canonical antioxidant response. Notably, SLE also increased the expression of p62/SQSTM1, which functions not only as a selective autophagy adaptor but also as a positive regulator of Nrf2 signaling through sequestration of Keap1 (33). These findings suggest that SLE may reinforce antioxidant responses through functional interaction between p62 and Nrf2 signaling pathways, thereby enhancing cellular resilience against oxidative stress. Such crosstalk between redox regulation and autophagy-related processes has emerged as an important determinant of neuronal survival under pathological conditions.

AMPK functions as a central metabolic sensor and has been increasingly implicated in neuroprotection through regulation of redox balance, mitochondrial function, and autophagy-related pathways (34). In the present study, SLE increased AMPK phosphorylation, and Compound C attenuated the SLE-induced increase in Nrf2 fluorescence. These findings suggest that AMPK activation may be involved, at least in part, in the regulation of Nrf2 nuclear localization in response to SLE treatment. Mechanistically, AMPK has been reported to promote Nrf2 activation through direct phosphorylation and/or modulation of upstream kinases, thereby enhancing Nrf2 stability and transcriptional activity (35). Collectively, these observations support a model in which SLE activates an AMPK-dependent redox regulatory network linking energy sensing to antioxidant defense.

Beyond redox regulation, mitochondrial homeostasis is a critical determinant of neuronal survival in PD, and impairment of mitochondrial turnover contributes to the accumulation of dysfunctional mitochondria and neuronal damage (36). In this study, SLE altered several mitophagy-related markers in a time-dependent manner. Early-phase responses included reductions in Parkin and phosphorylated p62 levels, accompanied by a modest increase in PINK1 expression, whereas later-phase responses involved an increased LC3-II/LC3-I ratio and decreased p62 protein levels. These changes were associated with restoration of mitochondrial membrane potential. Although these findings are consistent with changes in mitochondrial homeostasis-related processes, mitophagic flux was not directly assessed, and the subcellular localization of PINK1 and Parkin was not examined. Therefore, whether SLE directly regulates mitophagy or more broadly affects mitochondrial homeostasis remains to be clarified (37, 38). Further studies using mitophagy flux analyses, genetic approaches, and mitochondrial functional assays are required to elucidate the precise mechanisms involved.

Mitochondrial dysfunction and oxidative stress converge to trigger apoptotic cell death in dopaminergic neurons (39). In the present study, SLE attenuated rotenone-induced nuclear morphological changes associated with apoptosis, suggesting that suppression of cell death pathways contributes to its protective effects. Given the established interplay between mitochondrial integrity, ROS production, and apoptotic signaling, it is plausible that the observed anti-apoptotic effects of SLE are secondary to its upstream actions on redox regulation and mitochondrial homeostasis. Further studies employing specific apoptotic markers, including caspase activation and Bcl-2 family proteins, will be required to clarify these mechanisms in greater detail.

Importantly, the protective effects of SLE observed *in vitro* were partially corroborated *in vivo* using a rotenone-induced PD mouse model. SLE administration improved or tended to improve motor performance in rotarod, pole, and wire hang tests and attenuated the rotenone-induced reduction in TH immunoreactivity in the substantia nigra. These findings suggest that SLE may counteract rotenone-induced alterations in the nigrostriatal dopaminergic system *in vivo*, supporting its potential relevance as a food-derived preventive approach for PD-related neurodegenerative processes (40, 41). However, because the present histological analysis was based on TH staining intensity rather than direct stereological counting of TH-positive neurons, the observed preservation of TH immunoreactivity cannot be interpreted as definitive evidence of increased dopaminergic neuronal survival. Future studies employing unbiased stereological cell counting together with neuronal markers will be required to determine whether SLE directly prevents dopaminergic neuronal loss.

Based on these findings, we propose that SLE protects dopaminergic neuronal cells against rotenone-induced injury through activation of the AMPK–Nrf2 antioxidant pathway and modulation of mitochondrial homeostasis-related processes. As summarized in graphical abstract, SLE-induced AMPK activation may promote Nrf2 nuclear translocation, leading to upregulation of antioxidant genes such as HO-1, while p62/SQSTM1 may further reinforce Nrf2 signaling through interaction with Keap1. In parallel, SLE may support mitochondrial quality control through regulation of PINK1/Parkin/p62/LC3-related pathways, thereby reducing ROS accumulation, restoring mitochondrial membrane potential, and attenuating neuronal cell death.

A critical consideration for translational application is the bioavailability and brain accessibility of SLE-derived bioactive compounds. Although the ability of intact SLE components to cross the blood–brain barrier was not directly assessed in this study, accumulating evidence indicates that certain flavonoids and their metabolites can penetrate the blood–brain barrier and exert neuroprotective effects (42, 43). Therefore, it is plausible that bioactive constituents of SLE, or their metabolites, contribute to the observed *in vivo* efficacy. Future studies aimed at identifying active components and characterizing their pharmacokinetic properties will be essential for advancing the translational potential of SLE.

From a nutritional and translational perspective, the present findings highlight the potential value of strawberry leaves as a polyphenol-rich agricultural by-product with possible application as a nutraceutical ingredient or functional food component. Strawberry leaves are generally underutilized or discarded during fruit production and processing, although they contain diverse phenolic constituents, including ellagitannins, proanthocyanidins, flavonols, phenolic acids, and quercetin- and kaempferol-derived compounds. This multi-target mode of action may be advantageous in complex neurodegenerative disorders such as PD, in which oxidative stress, mitochondrial dysfunction, and dopaminergic neuronal loss are closely interconnected (44, 45). Thus, SLE may represent a sustainable, value-added food-derived material for supporting neuronal resilience against neurodegenerative stress. Further studies are required to establish its phytochemical standardization, safety, and efficacy in humans.

Despite these promising findings, a limitation of the present study is that detailed phytochemical profiling of SLE was not performed. Although previous studies have demonstrated that strawberry leaves contain a wide variety of phenolic compounds, the specific constituents responsible for the neuroprotective effects observed in the present study remain to be identified. An additional limitation is that total polyphenol content provides only an overall estimate of phenolic abundance and does not provide information on the qualitative or quantitative composition of individual phenolic constituents. Thus, extracts with similar total polyphenol contents may nevertheless differ substantially in their phytochemical profiles and biological activities. Future studies using analytical approaches such as HPLC-DAD and LC–MS/MS will therefore be important to characterize the major phenolic constituents of SLE and to determine which individual compounds or combinations of compounds contribute to its protective effects.

## 5 Conclusion

In conclusion, the present study demonstrates that SLE exerts neuroprotective effects through activation of the AMPK–Nrf2 signaling axis and regulation of mitochondrial homeostasis-related pathways. These effects were associated with suppression of oxidative stress, improvement of mitochondrial function, and attenuation of neuronal damage in both *in vitro* and *in vivo* models of PD.

These findings provide mechanistic insight into the potential of SLE as a food-derived approach for supporting neuronal resilience against neurodegenerative stress. Further studies are required to identify the active components, clarify their pharmacokinetic properties, and establish their translational potential.

## Data Availability

The raw data supporting the conclusions of this article will be made available by the authors, without undue reservation.
