# Supplementary material for: Strawberry leaf extract protects dopaminergic neurons by restoring redox and mitochondrial homeostasis
Source: Front Nutr. 2026 Sep 7;13:1893670. doi: 10.3389/fnut.2026.1893670 (PMC13590712; doi:10.3389/fnut.2026.1893670)
Supplement: Supplementary file 1 [file Supplementary_file_1.docx]

Supplementary Materials

**Supplementary Figure 1.** Non-adjusted full images of immunoblots in Fig. 6. Images were obtained using AE-9300 Ez-Capture MG (ATTO).

**Supplementary Figure 2.** Non-adjusted full images of immunoblots in Fig. 8(A). Images were obtained using AE-9300 Ez-Capture MG (ATTO).

**Supplementary Figure 3.** Non-adjusted full images of immunoblots in Fig. 8(B). Images were obtained using AE-9300 Ez-Capture MG (ATTO).

**Supplementary Figure 4.** Non-adjusted full images of immunoblots in Fig. 8(D). Images were obtained using AE-9300 Ez-Capture MG (ATTO).

**Supplementary Figure 5.** Non-adjusted full images of immunoblots in Fig. 8(E). Images were obtained using AE-9300 Ez-Capture MG (ATTO).

**Supplementary Figure 6.** Non-adjusted full images of immunoblots in Fig. 8(G). Images were obtained using AE-9300 Ez-Capture MG (ATTO).
